# Ultrasound cardiography examinations detect victims’ long-term realized and potential consequences after major disasters: a case-control study

**DOI:** 10.1186/s12199-018-0721-4

**Published:** 2018-08-14

**Authors:** Hidenori Onishi, Osamu Yamamura, Shinsaku Ueda, Muneichi Shibata, Soichi Enomoto, Fumie Maeda, Hiromasa Tsubouchi, Takeshi Hirobe, Sadao Shimizu, Kazuhiko Hanzawa, Tadanori Hamano, Yasunari Nakamoto, Hiroyuki Hayashi, Hidekazu Terasawa

**Affiliations:** 1 Department of Medical Technology, Kitasato Junior College of Health and Hygienic Sciences, Niigata, Japan; 2 Department of General Medicine, University of Fukui Hospital, Fukui, Japan; 3 Department of Community Medicine, Faculty of Medical Science, University of Fukui, 23-3 Matsuokashimoaizuki Yoshida-gun Eiheiji-cho, Fukui, 910-1104 Japan; 4 Second Department of Internal Medicine, Faculty of Medical Science, University of Fukui, 23-3 Matsuokashimoaizuki Yoshida-gun Eiheiji-cho, Fukui, 910-1104 Japan; 5 Department of Thoracic Surgery, Ishinomaki Red Cross Hospital, Miyagi, Japan; 6 Department of Cardiology, Makabe Hospital, Miyagi, Japan; 7 Department of Clinical Laboratory, University of Fukui Hospital, Fukui, Japan; 8 Department of Radiotechnology, Fukui Ken Saiseikai Hospital, Fukui, Japan; 9 Department of Clinical Laboratory, Fukui Prefectural Hospital, Fukui, Japan; 10 Department of Research Laboratory, National Hospital Organization Awara Hospital, Fukui, Japan; 11 Department of Respiratory Surgery, Graduate School of Medicine, University of Niigata, Niigata, Japan

**Keywords:** Great East Japan Earthquake, Tsunami disaster area, Temporary housing, Ultrasound cardiography examination, Disaster-related diseases

## Abstract

**Background:**

An increase in cardiovascular diseases has been reported following major disasters. Previous work has shown that ultrasonographic findings from ultrasound cardiography examination (UCG) increased until the 44th month after the tsunami caused by the Great East Japan Earthquake. The present study conducted UCG among victims in the tsunami disaster area and investigated the frequency of disaster-related cardiovascular diseases and changes over time until the 55th month after the disaster.

**Methods:**

The subjects were residents of temporary housing complexes and neighboring housing in Watari-gun, Miyagi Prefecture, Japan. There were 207 subjects in the 18th month, 125 in the 30th month, 121 in the 44th month, and 106 in the 55th month after the disaster. Data were collected through UCG and self-report questionnaire.

**Results:**

Significant changes were observed among subjects with clinical findings from the UCG, which increased over the study period—from 42.0 to 60.8, 72.7, and 73.6% beginning in the 18th month after the disaster (*p* < 0.0001).

**Conclusions:**

It is possible that the UCG can become a useful examination to visualize the potential impact of a major disaster on the cardiac function of victims. Victims with clinical findings continued increasing not only during the acute phase after a disaster but also in the long term. We therefore need to keep this in mind, and note that it is important to establish a support system to control cardiovascular diseases from the early stage of disaster.

**Trial registration:**

UMIN; ID000029802. R000034050. 2 November 2017.

## Background

Increases in disaster-related diseases and deaths due to degradation of the living environment have been reported in areas located near major disasters. Cardiovascular disease is an example of a disaster-related disease, as came to be noted in Japan when the number of deaths caused by acute myocardial infarction increased following the Great Hanshin-Awaji Earthquake (1995) [1]. An increase in takotsubo cardiomyopathy due to stress was confirmed after the subsequent Niigata-Chuetsu Earthquake (2004). After the Noto Hanto Oki Earthquake (2007), most cases of acute coronary syndrome were confirmed within 7 days of the disaster [2, 3]. Additionally, in the case of the Great East Japan Earthquake (2011), there was an increase in arrhythmia, as well as an increase in heart failure [4]. In our previous study investigating the tsunami disaster area in Watari-gun, Miyagi Prefecture, we found that the number of clinical findings from cardiac ultrasound examination increased every year until the 44th month after the disaster [5]. However, existing work has not yet clarified changes over time in cardiovascular diseases among disaster victims. In the present study, we conducted ultrasound cardiography examinations (UCG) among victims in Watari-gun, Miyagi Prefecture, the area affected by the tsunami caused by the Great East Japan Earthquake. Based on the clinical findings obtained, we then investigated the frequency of cardiovascular diseases occurring as disaster-related diseases and their change over time until the 55th month after the disaster.

## Methods

### The state of damage at the site

**Fig. 1:**
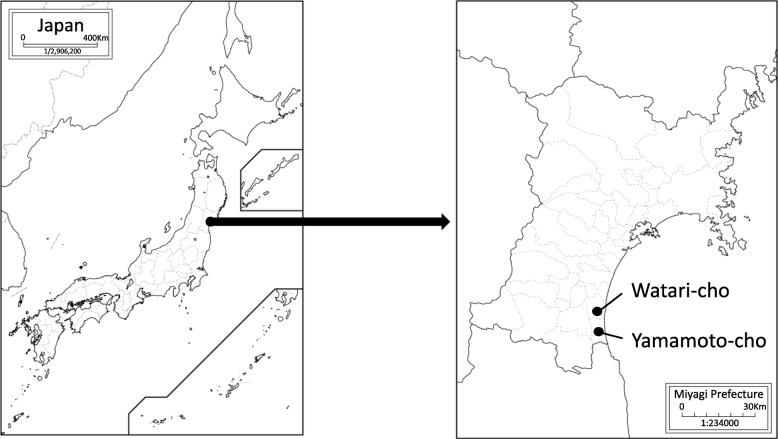
Research area: Watari-gun, Miyagi Prefecture (Watari-cho and Yamamoto-cho), is located approximately 40 km south of Sendai City. Watari-gun is an agricultural and fishery industrial area with a population of approximately 50,000

Watari-gun, Miyagi Prefecture (Watari-cho and Yamamoto-cho), is located approximately 40 km south of Sendai City. Watari-gun is an agricultural and fishery industrial area with a population of approximately 50,000 (Fig. 1). Regarding the human damage caused by the Great East Japan Earthquake, 885 died or are missing, there were up to approximately 13,000 evacuees, and 7075 houses were completely or partially destroyed. In September 2011, the number of victims living in temporary housing complexes reached 6050. As of May 2017, these complexes in Watari-gun were mostly removed.

### Subjects

In our investigation which we conducted four times, we examined 922 victims who were residents of the temporary housing complexes and neighboring area in Watari-gun, Miyagi Prefecture. Of these subjects, we excluded 55 for whom we found inadequacies in the medical questionnaire form, as well as 308 re-examinees. Also, the subjects for each year were limited only to new examinees. The final number of subjects was 559: 207 in the 18th month after the disaster (45 men, 162 women; mean age = 70.2 ± 9.9 years), 125 in the 30th month after the disaster (37 men, 88 women; mean age = 71.4 ± 9.9 years), 121 in the 44th month after the disaster (32 men, 89 women; mean age = 71.2 ± 7.6 years), and 106 in the 55th month after the disaster (35 men, 71 women; mean age = 66.6 ± 13.2 years).

### Method of ultrasonic examination: medical examination venue and the examining organization

Examinations were conducted at meeting places in temporary housing complexes. There were seven sites in Yamamoto-cho and five in Watari-cho, Watari-gun, Miyagi Prefecture. We conducted examinations for three consecutive years at six of these sites. Because the removal of the temporary housing complexes had progressed, beginning in the 44th month, we used the health center instead in Yamamoto-cho. We also used public spaces such as a central hall, Arahama Community Hall, and Yoshida Community Hall beginning in the 55th month in Watari-cho. The medical examination team was organized mainly by Fukui University Hospital, with cooperation from volunteer doctors, nurses, medical radiology technologists, laboratory medical technologists, occupational therapists, medical students, dental students, and nursing students.

### Medical examination items

After obtaining written consent from all of the subjects, medical examinations were conducted in the following order: a medical questionnaire form, blood pressure measurement, ultrasound examination, and the explanation of the results. The questionnaire form collected information on the subjects’ age, sex, past medical history, subjective symptoms, and lifestyle (smoking and exercise habits). The blood pressure evaluation was classified into four factors (systolic blood pressure, diastolic blood pressure, pulse pressure, and mean blood pressure). For the UCG, we used ultrasound equipment with high portability [6]. Regarding the evaluation of the UCG, we visually examined the following as we emphasized time efficiency: the size of the atrium/ventricle, vascular diameter (diameter of the aorta and inferior vena cava), left ventricular wall thickness, left ventricular wall motion, valve property, and regurgitation. We regarded other findings as special-mention items. Because a previous report indicated that the reliability and reproducibility of the left ventricular ejection fraction (LVEF) are the same whether applying a visual or a quantitative evaluation using the modified Simpson method, we applied a qualitative visual evaluation [7]. Visual evaluation is useful for grasping the disease condition even in focused cardiac ultrasound, and it was therefore adopted in the present study [8]. The ultrasound examination was conducted by an ultrasonographer with more than 5 years of experience who was certified by The Japan Society of Ultrasonics in Medicine. For portable ultrasound equipment, we used the Noblus (Hitachi Medical Corporation, frequency band 1–5 MHz sector probe), the CX 50 (Philips Electronics Japan, frequency band 1–5 MHz sector probe), the LOGIQ e (GE Healthcare, frequency band 1.5–4.0 MHz sector probe), and the Viamo (Toshiba Medical Systems, frequency band 1.8–4.2 MHz sector probe). We defined atrioventricular dilatation, vascular dilatation, left ventricular wall hypertrophy, left ventricular asynergy, LVEF < 40%, and valvular disease (including mild cases, regurgitation with high–normal blood pressure, and calcified lesions) as clinical findings; other findings were defined as special-mention items [9].

### Statistical analysis

Age, systolic blood pressure, diastolic blood pressure, pulse pressure and mean blood pressure values are presented as means ± standard deviations. Nominal variables are presented as the frequency (%) of cases for each item. We used Rcommander Version 1.28 for the statistical analysis. We used the Mann–Whitney *U* test and the *χ*^2^ test (including Yates consecutive correction) for comparisons between two groups, and we used ANOVA (one-way analysis of variance) and Fisher’s exact test for comparisons among three groups. The Cochran–Armitage test was used to test trends. Multiple logistic regression analysis (stepwise method) was used to detect factors determining the clinical findings. In all comparative tests, a *p* value of < 0.05 was defined as significant.

## Results

### Changes over time in subjects’ backgrounds

**Fig. 2:**
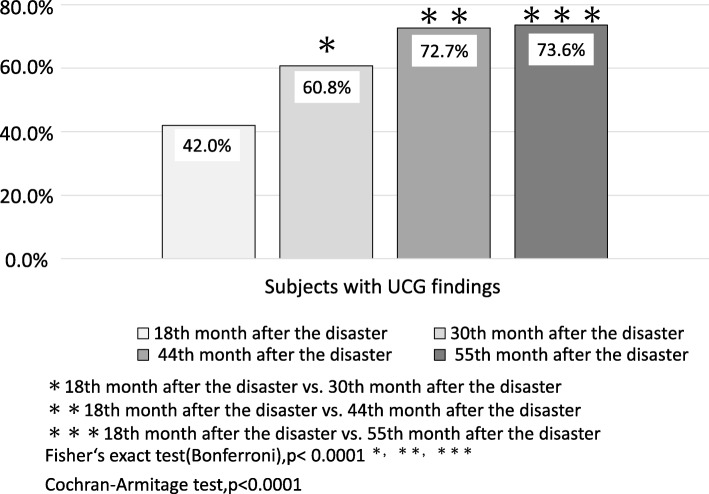
Comparison of the ultrasound cardiography (UCG) findings in each year: The number of people with clinical findings significantly increased from the 18th month to the 30th month, from the 18th month to the 44th month, and from the 18th month to the 55th month after the disaster (*p* < 0.0001). The trend test also showed an increasing trend in the findings (*p* < 0.0001)

The UCG screening was conducted once a year for 4 years, and 559 people (149 men, 410, women; mean age = 70.0 ± 10.3 years) were examined. Of these, there were clinical findings for 329 people (54.9%). Regarding changes over time, significant changes in age were observed from the 18th to the 30th month after the disaster, from the 30th to the 55th month after the disaster, and from the 44th to the 55th month after the disaster (*p* < 0.01). As for diastolic blood pressure, a significant change was observed from the 18th month to the 30th month after the disaster (*p* < 0.05). The number of people with clinical findings significantly increased from the 18th month to the 30th month, from the 18th month to the 44th month, and from the 18th month to the 55th month after the disaster (*p* < 0.0001) (Fig. 2). The trend test also showed an increasing trend in the findings (*p* < 0.0001). Dyslipidemia, which was the underlying disease, significantly increased from the 18th month to the 30th month and from the 18th month to the 44th month after the disaster (*p* < 0.001). As for the living environment after the disaster, the number of people who were living in temporary housing complexes decreased each year, with an especially large decrease after the 44th month following the disaster (*p* < 0.0001). There was no significant change in other items (Table 1).

**Table 1:** Changes over time in subjects’ backgrounds

	18th month after the disaster	30th month after the disaster	44th month after the disaster	55th month after the disaster	Total	*p* value
	*n* = 207	*n* = 125	*n* = 121	*n* = 106	*n* = 559	
Age (years)	70.2 ± 9.9	71.4 ± 9.9	71.2 ± 7.6	66.8 ± 13.2	70.0 ± 10.3	< 0.01 ^cef^
Gender (male/female)	45/162	37/88	32/89	35/71	149/410	ns
Blood pressure						
SBP (mmHg)	138.7 ± 18.4	135.4 ± 18.2	139.1 ± 18.3	136.9 ± 18.9	137.7 ± 18.4	ns
DBP (mmHg)	82.8 ± 12.3	78.8 ± 11.7	81.5 ± 12.4	80.3 ± 12.8	81.1 ± 12.3	< 0.05^a^
PP (mmHg)	55.8 ± 14.1	56.5 ± 16.1	57.5 ± 15.0	56.6 ± 16.3	56.5 ± 15.2	ns
MBP(mmHg)	101.4 ± 13.0	97.7 ± 12.0	100.7 ± 12.8	99.2 ± 13.1	100.0 ± 12.8	ns
Subjects with UCG findings, *n* (%)	87 (42.0)	76 (60.8)	88 (72.7)	78 (73.6)	329 (58.8)	< 0.0001^abc^
Lifestyle						
Exercises, *n* (%)	121 (58.4)	81 (64.8)	82 (67.7)	55 (51.8)	339 (60.6)	ns
Smoker, *n* (%)	16 (7.7)	11 (8.8)	10 (8.2)	11 (10.3)	48 (8.0)	ns
Underlying disease						
DL, *n* (%)	68 (32.8)	65 (52.0)	63 (52.0)	48 (45.2)	244 (43.6)	< 0.001^ab^
DM, *n* (%)	23 (11.1)	21 (16.8)	19 (15.7)	17 (16.0)	80 (14.3)	ns
Heart disease, *n* (%)	54 (25.9)	37 (29.6)	38 (31.4)	29 (27.3)	158 (28.2)	ns
HT, *n* (%)	111 (53.6)	72 (57.6)	70 (57.8)	47 (44.3)	300 (53.6)	ns
Insomnia, *n* (%)	106 (51.2)	66 (52.8)	46 (38.0)	47 (44.3)	265 (47.4)	ns
Living environment						
Temporary housing resident, *n* (%)	199 (96.1)	114 (91.2)	50 (41.3)	23 (21.7)	386 (69.0)	< 0.0001^bcdef^

Mean ± standard deviation. Nominal variables are shown as frequency (%). Fisher’s exact test (Bonferroni) and ANOVA (Bonferroni). Heart disease (coronary artery disease, arrhythmia, heart failure, valve regurgitation). In our investigation which we conducted four times, we examined 922 victims who were residents of the temporary housing complexes and neighboring area in Watari-gun, Miyagi Prefecture. Of these subjects, we excluded 55 for whom we found inadequacies in the medical questionnaire form, as well as 308 re-examinees. Also, the subjects for each year were limited only to new examinees. The final number of subjects was 559

*ns* non-significant difference. *SBP* systolic blood pressure, *DBP* diastolic blood pressure, *PP* pulse pressure, *MBP* mean blood pressure, *UCG* ultrasound cardiography, *DL* dyslipidemia, *DM* diabetes mellitus, *HT* hypertension

^a^18th month after the disaster vs. 30th month after the disaster ^b^18th month after the disaster vs. 44th month after the disaster. ^c^18th month after the disaster vs. 55th month after the disaster. ^d^30th month after the disaster vs. 44th month after the disaster. ^e^30th month after the disaster vs. 55th month after the disaster. ^f^44th month after the disaster vs. 55th month after the disaster

There were many cases of mild valve regurgitation in the clinical findings (Table 2).

**Table 2:** Observed items from UCG findings

	18th month after the disaster	30th month after the disaster	44th month after the disaster	55th month after the disaster
	*n* = 87	*n* = 76	*n* = 88	*n* = 78
Left atrial dilatation	9 (10.3)	35 (26.0)	28 (31.8)	15 (19.2)
Other atrioventricular dilatation	5 (5.7)	3 (3.9)	4 (4.5)	1 (1.3)
Vascular diameter (aorta diameter or inferior vena cava)	1 (1.1)	8 (10.5)	1 (1.1)	2 (2.6)
Left ventricular wall thickness	10 (11.4)	2 (2.6)	22 (25.0)	3 (3.8)
Left ventricular asynergy	1 (1.1)	7 (9.2)	5 (5.7)	3 (3.8)
Reduction in left ventricular ejection fraction	2 (2.3)	9 (11.8)	5 (5.7)	1 (1.3)
Pericardial effusion	1 (1.1)	1 (1.3)	3 (3.4)	1 (1.3)
Interatrial septal aneurysm	1 (1.1)	1 (1.3)	0 (0)	0 (0)
Aortic valve stenosis	3 (3.4)	7 (9.2)	2 (2.3)	3 (3.8)
Aortic valve stenosis (moderate or higher)	0 (0)	0 (0)	1 (1.1)	0 (0)
Aortic valve regurgitation	31 (35.6)	27 (35.5)	41 (46.5)	35 (44.9)
Aortic valve regurgitation (moderate or higher)	5 (5.7)	2 (2.6)	18 (15.8)	5 (6.4)
Aortic valve calcification	31 (35.6)	11 (14.4)	5 (5.7)	4 (5.1)
Aortic valve thickening	3 (3.4)	3 (3.9)	0 (0)	0 (0)
Mitral valve stenosis	0 (0)	3 (3.9)	1 (1.1)	3 (3.8)
Mitral valve stenosis (moderate or higher)	0 (0)	0 (0)	0 (0)	0 (0)
Mitral valve regurgitation	28 (32.1)	25 (32.9)	56 (63.6)	58 (74.3)
Mitral valve regurgitation (moderate or higher)	4 (4.6)	4 (5.3)	13 (14.8)	3 (3.8)
Mitral valve calcification	8 (9.2)	7 (9.2)	2 (2.3)	2 (2.6)
Mitral valve prolapse	0 (0)	1 (1.3)	2 (2.3)	0 (0)
Tricuspid valve regurgitation	6 (9.9)	16 (21.0)	16 (18.2)	31 (39.7)
Tricuspid valve regurgitation (moderate or higher)	2 (2.3)	7 (9.2)	10 (11.3)	3 (3.8)
Pulmonic valve regurgitation	1 (1.1)	3 (3.9)	1 (1.1)	10 (12.8)
Pulmonic valve regurgitation (moderate or higher)	1 (1.1)	1 (1.3)	0 (0)	0 (0)

Nominal variables are shown as frequency (%), including compound examples

*UCG* ultrasound cardiography

### Comparison of the UCG findings

We compared the group with clinical findings (329 people) with the group with no clinical findings (230 people). Those with clinical findings were significantly older (71.8 ± 9.5 years) than were those with no findings (67.6 ± 10.9 years) (*p* < 0.0001). Systolic blood pressure was significantly higher in the group with findings (139.2 ± 18.5 mmHg), compared with the group with no findings (135.5 ± 18.2 mmHg) (*p* < 0.05). Pulse pressure was significantly higher in the group with findings (58.5 ± 16.0 mmHg) than in the group with no findings (53.6 ± 13.5 mmHg) (*p* < 0.001). There were significantly more subjects who exercised (65.3%) in the group with findings than in the group with no findings (53.9%) (*p* < 0.01). Heart disease was significantly more common in the group with findings: There were 107 people (32.5%) with heart disease in this group, compared with 51 in the group with no findings (22.2%) (*p* < 0.01) (Table 3).

**Table 3:** Comparison by clinical findings (all 559 cases)

	Findings	No findings	*p* value
	*n* = 329	*n* = 230	
Age (years)	71.8 ± 9.5	67.6 ± 10.9	< 0.0001
Gender (male/female)	235/94	175/55	ns
Blood pressure			
SBP (mmHg)	139.2 ± 18.5	135.5 ± 18.2	< 0.05
DBP (mmHg)	80.7 ± 12.2	81.8 ± 12.6	ns
PP (mmHg)	58.5 ± 16.0	53.6 ± 13.5	< 0.001
MBP (mmHg)	100.2 ± 12.5	99.7 ± 13.3	ns
Lifestyle			
Exercises, *n* (%)	215 (65.3)	124 (53.9)	< 0.01
Smoker, *n* (%)	26(7.9)	22 (8.6)	ns
Underlying disease			
DL, *n* (%)	152 (46.2)	92 (40.0)	ns
DM, *n* (%)	47(14.3)	33 (14.3)	ns
Heart disease, *n* (%)	107 (32.5)	51(22.2)	< 0.01
HT, *n* (%)	182 (55.3)	118 (51.3)	ns
Insomnia, *n* (%)	153 (46.5)	112 (48.7)	ns
Living environment			
Temporary housing resident, *n* (%)	214 (65.0)	172 (74.8)	< 0.05

Mean ± standard deviation. Nominal variables are shown as frequency (%). Mann–Whitney *U* test, *χ*2 test, multiple logistic regression analysis. Heart disease (coronary artery disease, arrhythmia, heart failure, valve regurgitation)

*ns* non-significant difference, *SBP* systolic blood pressure, *DBP* diastolic blood pressure, *PP* pulse pressure, *MBP* mean blood pressure *DL* dyslipidemia, *DM* diabetes mellitus, *HT* hypertension

Pooling the 4 years of study, the factors determining the UCG findings were age (*p* < 0.001), pulse pressure (*p* < 0.05), heart disease (*p* < 0.05), being in the 30th month after the disaster (*p* < 0.001), being in the 44th month after the disaster (*p* < 0.0001), and being in the 55th month after the disaster (*p* < 0.0001) (Table 4).

**Table 4:** Factors determining the UCG findings (all 559 cases)

	Odds ratio	Lower 95% CI	Upper 95% CI	*p* value
Age	1.04	1.02	1.06	< 0.0001
Male	1.2	0.788	1.83	ns
PP	1.02	1	1.03	< 0.05
Exercises	1.33	0.899	1.96	ns
Smoker	1.1	0.551	2.18	ns
DL	1.18	0.802	1.75	ns
DM	0.726	0.43	1.23	ns
Heart disease	1.53	1.01	2.32	< 0.05
HT	0.806	0.542	1.2	ns
Insomnia	0.988	0.676	1.44	ns
Temporary housing resident	1.61	0.907	2.87	ns
18th month after the disaster vs 30th month after the disaster	2.11	1.32	3.38	< 0.01
18th month after the disaster vs 44th month after the disaster	3.63	2.2	5.98	< 0.0001
18th month after the disaster vs 55th month after the disaster	4.98	2.86	8.67	< 0.0001

Heart disease (coronary artery disease, arrhythmia, heart failure, valve regurgitation)

*ns* non-significant difference, multiple logistic regression analysis (stepwise method)

*UCG* ultrasound cardiography, *PP* pulse pressure, *DL* dyslipidemia, *DM* diabetes mellitus, *HT* hypertension

## Discussion

Although the impact of major disasters on victims has been studied from various viewpoints, no previous studies have focused on the cardiac function. In the present study, we conducted UCG over a number of years for victims who were not patients at any healthcare facility, and identified the long-term impact on victims of a major disaster for the first time [5]. This study examination can serve as a useful reference when discussing mid-to-long-term post-disaster medical care in the future.

In the present study, the number of people with UCG findings increased until the 44th month after the disaster [5] but then remained stable in the 55th month after the disaster. However, the cases with increased findings were mainly mild ones with less morbid findings. This shows that the potential impact of a major disaster persists even during the disaster recovery phase. Medical examinations, electrocardiograms, and chest X-rays conducted during general checkups do not show any findings until diseases have developed. Because UCG detected minor changes before the onset of cardiac diseases, it is a useful examination to visualize the potential impact of a major disaster on victims.

Environmental changes for the subjects after the disaster can be identified from the background factors included in this study. First, one factor is the decrease in the number of people living in temporary housing that occurred from the 30th to the 44th month after the disaster. In the 55th month, the ratio of temporary housing residents decreased to a low of 21.7%. This shows that the living environment of the subjects changed significantly. Second, dyslipidemia increased significantly from the 18th month to the 30th month. Because over 90% of the residents were in temporary housing during this period, we consider the reason for the increase in dyslipidemia to be the change in diet after moving into temporary housing. It is possible that the increase in the group with findings up till the 44th month was due to the environmental change after the disaster. Additionally, in the multivariate analysis, it was shown that aging, pulse pressure, and course of time were independent risk factors for the group with clinical findings. Especially in the 55th month, despite the average age of the subjects being lower than the past three waves of data collection, the ratio of subjects with clinical findings was significantly higher. This shows that the passage of time after the disaster contributed more to the group with clinical findings than did the subjects’ age.

In this study, an increase in pulse pressure and the passage of time were found to be risk factors for the group with clinical findings. Pulse pressure is regarded as a risk factor for cardiovascular diseases because it indicates the hardening of elastic arteries and an abnormality in cardiac ejection volume. It is possible that, because an increase in pulse pressure promotes heart overload and causes outcomes such as left atrial enlargement, left ventricular hypertrophy, and left ventricular dilatation, its increase over the long term affected the UCG findings [10–14]. Nakamura M et al. reported that heart failure increased over several weeks after the disaster in the coastal area where it was assumed the victims had higher stress due to the tsunami damage, such as a loss experience. Also, Nakamura A et al. reported that the new-onset heart failure increased among inpatients in the disaster area. In particular, Nakamura A et al. pointed out that there were many cases of heart failure with preserved ejection fraction (HFpEF) as an increase factor [15, 16]. As for an increase factor for the clinical findings in the tsunami disaster area, it is possible that the HFpEF preliminary group was affected by the environmental change and stress of living in the temporary housing due to the tsunami disaster, and heart failure therefore became evident. The left atrial enlargement increased each year in comparison with that of the 18th month after the disaster and peaked at the 44th month after the disaster. Pritchett AM et al. also reported that the left atrial enlargement reflected the chronic left atrial overload due to the diastolic dysfunction [17]. Among the group with clinical findings in the present study, we can assume that the diastolic dysfunction caused by pulse pressure increase, resulted in having the chronic left atrial overload and then enlarged. Although we cannot directly assess the diastolic dysfunction, there is a report that indicated a correlation between the left atrial enlargement and diastolic dysfunction. It is therefore possible that the HFpEF increased in the present study. We can also assume that the mitral regurgitation increased over time due to the left atrial enlargement. Additionally, the average age of the subjects in our study was 70.8 ± 9.3 years, and elderly people accounted for the majority of the sample. This indicates the importance of mental health management for the elderly in the chronic phase of post-disaster medical care. It also suggests that a strong stress reaction, such as survivor’s guilt, can cause cognitive decline, disuse syndrome, and other problems [18–20]. Such stress is assumed to be difficult to improve through changes in the living environment.

Preventing the onset of cardiovascular disease is an important issue in disaster medical care. Kario, et al. suggests the introduction of disaster cardiovascular prevention (DCAP) risk score / prevention score and disaster cardiovascular prevention network systems [21, 22]. The DCAP risk score makes a significant contribution to detect high-risk patients with cardiovascular disease. Risk items are: 1. Age, 2. Family, 3. House, 4. Community, 5. Hypertension, 6. Diabetes, 7. Medical history of cardiovascular disease. Each item is one point so that it is evaluated with a total of seven points. Any patient with a score of four points or more is categorized to be a high-risk cardiovascular disease patient. Also, any victim with the risk score of four points or more should make an effort to raise the DCAP prevention score to more than six, on a basis of either an evacuation center or individual. DCAP prevention score items are: 1. Sleep, 2. Exercise, 3. Diet, 4. Weigh, 5. Infection, 6. Thrombosis, 7. Medication and 8. Pressure. Each item is one point so that it is evaluated with a total of eight points. Using both types of score is recommended for the disaster medicine to prevent the onset of cardiovascular disease in the acute phase. On the other hand, UCG is a useful examination to visualize the potential impact of the cardiac function, but it is difficult to conduct this for all the victims. Although we did not perform an assessment in the present study, a UCG examination should be conducted for high-risk groups detected by the DCAP score in the stage when the victims are living in temporary housing complexes. A remarkable increase in the group with clinical findings continued until the 44th month after the disaster in the present study. We therefore think that we should actively conduct preventive measures and medical checkups against cardiovascular disease after they have moved to temporary housing. A long-term support system is necessary to prevent the increase of cardiovascular diseases, while government, local medical institutions, and medical support teams from outside the affected areas cooperate together and help functional recovery of local medical institutions.

The data in this study were collected after the population had moved into temporary housing, and we did not compare our findings with those from data collected before the disaster or at the stage of living in shelters. For this reason, the impact of a major disaster itself on victims is not taken into account. Findings in the present study cannot be specific for the disaster because of a lack of data before the disaster. It is however possible that narrow and unfamiliar housing environments like temporary housing complexes contributed to the change.

## Conclusions

It is possible that the UCG can become a useful examination to visualize the potential impact of a major disaster on the cardiac function of victims. Victims with clinical findings continued increasing not only during the acute phase after a disaster but also in the long term. We therefore need to keep this in mind, and note that it is important to establish a support system to control cardiovascular diseases from the early stage of disaster.

## Abbreviations

DBP: Diastolic blood pressure
DCAP: Disaster cardiovascular prevention
DL: Dyslipidemia
DM: Diabetes mellitus
HFpEF: Heart failure with preserved ejection fraction
HT: Hypertension
LVEF: Left ventricular ejection fraction
MBP: Mean blood pressure
PP: Pulse pressure
SBP: Systolic blood pressure
UCG: Ultrasound cardiography
